# Methane and nitrous oxide emission from different treatment units of municipal wastewater treatment plants in Southwest Germany

**DOI:** 10.1371/journal.pone.0209763

**Published:** 2019-01-04

**Authors:** Azzaya Tumendelger, Zeyad Alshboul, Andreas Lorke

**Affiliations:** 1 Institute of Chemistry and Chemical Technology, Mongolian Academy of Sciences, Bayanzurkh district, Ulaanbaatar, Mongolia; 2 Institute for Environmental Sciences, University of Koblenz-Landau, Landau, Germany; 3 Civil Engineering Department, Faculty of Engineering, Applied Science University, Amman, Jordan; Nederlands Instituut voor Ecologie, NETHERLANDS

## Abstract

We measured the atmospheric emission rates of methane (CH_4_) and nitrous oxide (N_2_O) in two wastewater treatment plants in Southwest Germany, which apply different treatment technologies. Dissolved gas concentrations and fluxes were measured during all processing steps as well as in the discharge receiving streams. N_2_O isotopocule analysis revealed that NH_2_OH oxidation during nitrification contributed 86–96% of the N_2_O production in the nitrification tank, whereas microbial denitrification was the main production pathway in the denitrification tank in a conventional activated sludge (CAS) system. During wastewater treatment using a modified Ludzack-Ettinger system (MLE) with energy recovery, N_2_O was predominantly produced by the NO_2_^-^ reduction by nitrifier-denitrification process. For both systems, N_2_O emissions were low, with emission factors of 0.008% and 0.001% for the MLE and the CAS system, respectively. In the effluent-receiving streams, bacterial denitrification and nitrification contributed nearly equally to N_2_O production. The CH_4_ emission from the MLE system was estimated as 118.1 g-C d^-1^, which corresponds to an emission factor of 0.004%, and was three times lower than the emission from the CAS system with 0.01%.

## Introduction

Sewage treatment is an important source of anthropogenic greenhouse gases with significant amounts of methane (CH_4_), nitrous oxide (N_2_O) and carbon dioxide (CO_2_) being released during wastewater treatment [1]. CO_2_ is produced both indirectly as a result of fossil fuel combustion for energy generation that is required for the operation of waste water treatment plants (WWTPs), and it is produced during the degradation of organic matter during the treatment process. While the latter emissions are considered as short-cycle CO_2_ that does not contribute to increasing atmospheric CO_2_ concentrations [1], the emissions of CH_4_ and N_2_O from WWTPs contribute to the anthropogenic increase of atmospheric greenhouse gas concentration.

Over a 100-year time span, CH_4_ has 34-fold a global warming potential compared to CO_2_ [2]. It is mainly generated in the sewer system [3], in the anaerobic treatment zone and during sludge treatment [4]. N_2_O is produced during the biological nitrogen removal processes via nitrification and denitrification. It is the most powerful gas that destroys the ozone layer [5,6] and it has global warming potential which is 265 times greater than that of CO_2_ [2].

CH_4_ and N_2_O contribute for 16% and 6.2% to the global anthropogenic greenhouse gas emission, respectively, with the remainder consisting of CO_2_ (76%) and fluorinated gases (2%) [2]. The IPCC classifies the global anthropogenic emission in seven sectors, one of them being waste and wastewater. The wastewater treatment sector is assumed to be responsible for 3.2% (CH_4_) and 4–5% (N_2_O) of the total anthropogenic emissions [5,7]. However, these assessments are associated with a high degree of uncertainty because microbial activities are sensitive to numerous variables of the actual treatment processes, such as dissolved oxygen (DO) concentration, pH, temperature, and substrate availability. Very few direct measurements of emission rates exist, which showed relatively large variations [1,4,8,9,10,11,12]. Daelman et al. [2012] found that about 1.13% of the incoming chemical oxygen demand (COD) in the wastewater is emitted as CH_4_ from a WWTP located in the Netherlands, while 1.7% of the nitrogen loading rate (NLR) from nitrifying reactors, 15% of the NLR in full-scale reactors, and 95% of the NLR in lab-scale bioreactors were converted to N_2_O.

In addition to in-plant emissions during the treatment process, WWTP export CH_4_ and N_2_O in dissolved form with effluent discharge. In a regional-scale study, Alshboul et al. [2016] observed enhanced CH_4_ concentrations and fluxes downstream of WWTP in small streams in Central Europe [13]. The annual mean CH_4_ concentration in the treatment plant effluents was correlated to the corresponding mean COD of the treated wastewater. Enhanced concentrations and fluxes of N_2_O from rivers near urban areas and downstream of WWTP have also been attributed to WWTP effluents [14, 15, 16].

To assess the anthropogenic influence on emission rates and to obtain reliable climate change projections of anthropogenic N_2_O and CH_4_ emissions, improved process-based understanding of the relevance of varying environmental conditions are required. Prior studies revealed that N_2_O production rates are predominantly affected by varying temperature and DO concentrations [15;17]. The relative importance of the different microbial production pathways to N_2_O was based on correlation analysis with DO, dissolved organic carbon (DOC), nitrate (NO_3_^-^), and dissolved nitrous oxide (DN_2_O).

Stable isotopocule ratios of N_2_O (^15^N and ^18^O) have been explored as a noninvasive method to assess N_2_O production and consumption mechanisms in WWTPs, as well as in rivers and streams which receive effluents [18]. The interpretation of bulk δ^15^N and δ^18^O of N_2_O, however, is challenging because of the dependency of δ^15^N and δ^18^O on the isotopic composition of the precursors (ammonium (NH_4_^+^) and NO_3_^-^) and the uncertainty of isotopic fractionation during various processes. It has been suggested that the intramolecular distribution of δ^15^N in the asymmetric N_2_O molecule could serve as a tool to discern various N_2_O production and consumption processes and can help to constrain the global N_2_O budget [19; 20]. The site-preference (SP) is defined as the difference between the N isotopic ratios of the central and the terminal N atom (δ^15^N^α^ and δ^15^N^β^ values). An advantage of using SP is that it is assumed to be independent on δ^15^N of the precursors [19, 20]. The SP of N_2_O differs for hydroxylamine (NH_2_OH) oxidation during nitrification and nitrite (NO_2_^-^) reduction during denitrification. This selective approach has been applied in several studies including full scale wastewater treatment and in-situ measurements of different N_2_O production pathways during wastewater treatment processes [21,22,23], however, to a very limited extent to inland waters [24].

This study was conducted (1) to quantify CH_4_ and N_2_O emissions from two different WWTPs located in southern Germany, (2) to analyze the N_2_O production pathways based on isotopocule ratios of N_2_O in these systems to parameterize isotopocule ratios of N_2_O emitted from WWTP, and (3) to determine the influence of N_2_O and CH_4_ discharge with WWTP effluent on concentrations and fluxes in the receiving streams.

## Materials and methods

### Study site

**Fig 1:**
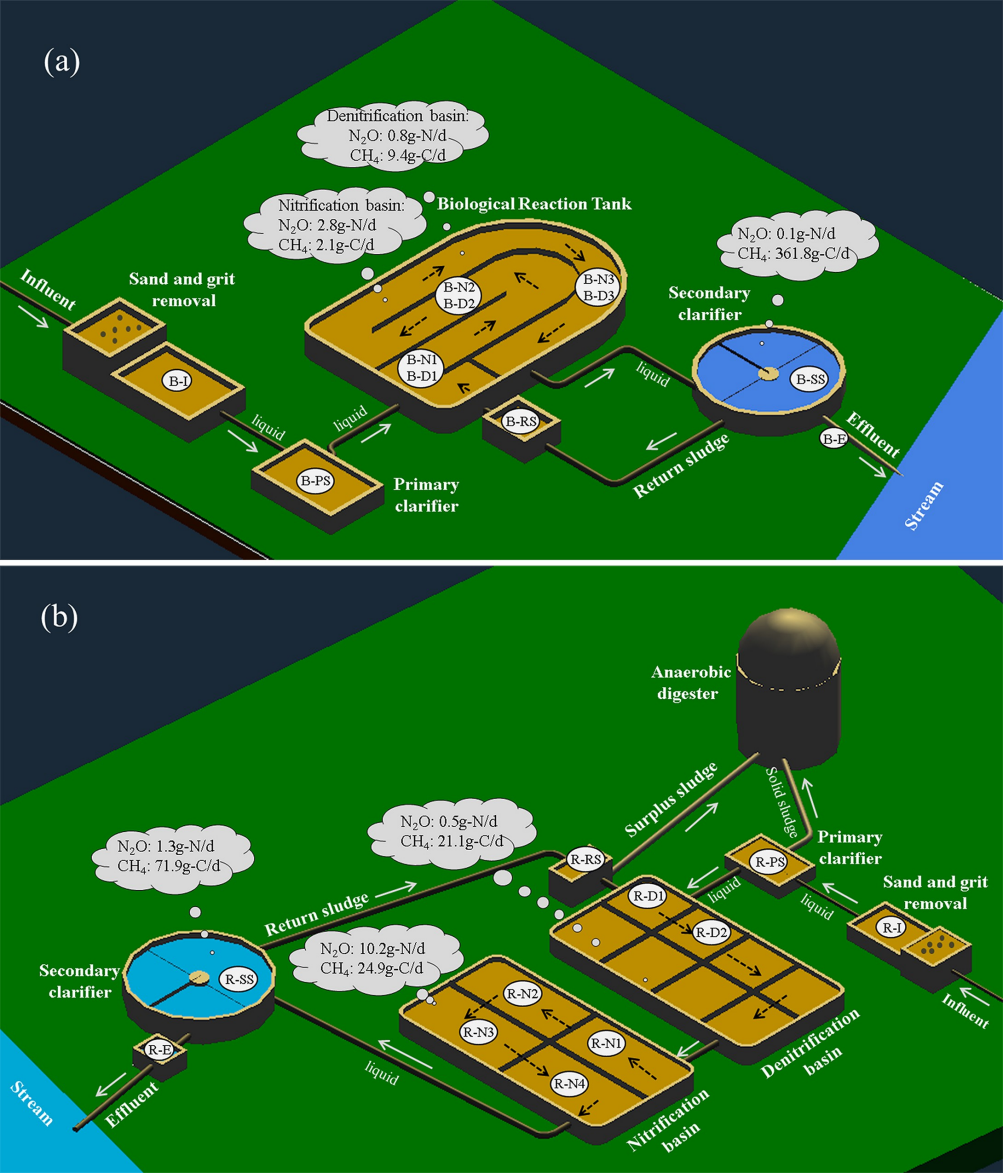
Process schematics of the wastewater treatment plants with estimated emission rates of N_2_O and CH_4_ from the units: (a) Conventional activated sludge system at Bellheim, (b) Modified Ludzack-Ettinger system with anaerobic digestion at Ruelzheim. Sampling stations are shown as ID in the light-gray circle.

Two municipal WWTPs located in Southwest Germany were studied in this investigation: Bellheim and Ruelzheim. The Bellheim WWTP, which receives wastewater from a population equivalent (PE) of 14.3 thousand at an average flow rate of approximately 5×10^3^ m^3^ day^-1^, applies a conventional activated sludge (CAS) treatment system. The system comprises two clarifiers and a series of biological reaction tanks. Heavy solids are removed from wastewater in the primary clarifier. The water then undergoes biological treatment to decompose organic matter by activated sludge under aerobic and anaerobic conditions. The respective switching time between denitrification and nitrification process were 45 and 80 min, respectively. Subsequently, the microbe-rich liquid flows into the secondary clarifier where activated sludge is separated from treated wastewater by gravity. Parts of the settled sludge is continuously recycled back to the aeration tanks to maintain the microbial community and the treated effluent is finally discharged into a neighboring stream (Fig 1A).

The treatment capacity of Ruelzheim WWTP is three times greater than Bellheim WWTP, and it applies a modified Ludzack-Ettinger (MLE) system with energy recovery. It treats wastewater from 41.5 thousand PE with a flow rate of approximately 14.7×10^3^ m^3^ day^-1^. The surplus sludge from the plant is treated in an anaerobic digester for biogas production. The gas is used for digester heating and electricity generation and provides about 60.8% of the total in-plant energy requirements. Wastewater treated by the primary clarifier enters an anaerobic tank for denitrification and then an aerobic tank for nitrification by activated sludge. From the aeration tank, the mixed liquor flows to the secondary clarifiers to separate treated wastewater from the sludge. Parts of the sludge are recycled back into the biological tank, while the remaining sludge is fed to the anaerobic digester for energy generation. The last step of this treatment system is identical to the CAS system (Fig 1B).

### Sample collection

Water samples were collected as duplicates at eleven sites in each treatment system (Fig 1, permissions were provided by the treatment plant operator): influent wastewater (R-I and B-I), outflow of the primary clarifier (R-PS and B-PS), nitrification / denitrification basins (sections R-D1, R-D2, R-N1‐R-N4 in Ruelzheim WWTP and sections B-N1, B-N2, B-N3 and zone B-D1, B-D2 and B-D3 in Bellheim WWTP), secondary clarifier, return sludge tunnel and effluent (exit of secondary clarifier, R-SS and B-SS). Additional samples were collected up- and downstream of the effluent discharge point in the receiving streams (no specific permission required). The downstream sampling sites in the receiving streams were located where effluent and stream water were well mixed (R-DS-M, B-DS-M) and additional samples were collected at 50 m distance from the effluent discharge (R-DS-50, B-DS-50).

Dissolved CH_4_ (DCH_4_) and CO_2_ (DCO_2_) concentrations in water samples were measured on-site using the headspace method. The headspace was created in a borosilicate glass bottle and the gas partial pressure was measured in a closed gas loop with an ultraportable greenhouse gas analyzer (*UGGA*, Los Gatos Research Inc.). More detailed information about the concentration measurements can be found in Alshboul et al. [2016]. DN_2_O concentrations were measured using a gas chromatograph equipped with an electron capture detector (ECD). The concentrations of dissolved NH_4_^+^, NO_3_^-^ and NO_2_^-^ were measured using a portable spectrophotometer (DR 2800™; Hach company, Colorado, US). Water samples for isotopic analyses were transferred into 250 mL glass vials without a headspace, sterilized with 5 mL of saturated HgCl_2_, sealed with butyl rubber stoppers and aluminum caps and stored at 4°C until analysis. Samples for concentration and isotope analysis of NH_4_^+^ and NO_3_^-^ were filtered into 50 mL plastic bottles and kept in a freezer at -35°C until analysis.

Water temperature, DO, pH, specific conductivity and redox potential were measured on site using a pH-temperature electrode with gel electrolyte (SenTix 21; 0–14 pH; 0–80°C; ±0.2°C), a DO sensor (FDO 925; 0–20 mg L^-1^ ± 0.5%; 0–50 ± 0.2°C), a conductivity cell (TetraCon 925, 10–2000 ± 0.5% mS cm^-1^; 0–100 ± 0.2°C) and an oxidation/reduction potential electrode (SenTex ORP 900; ± 1200.0 ± 0.2 mV; platinum) connected to a portable three channel multi meter (3430 IDS; WTW GmbH).

### Flux measurements

Fluxes of N_2_O and CH_4_ at the air-water interface in the treatment ponds and the effluent receiving streams were measured using floating chambers (surface area: 0.078 m^2^, chamber volume: 7.66 l), covered with aluminum foil to reduce the internal heating. The flux measurements were performed using triplicate floating chamber deployments with 35 min duration each. The slope describing the rate of change of the measured headspace concentration in the chamber over time was used to calculate the CH_4_ and N_2_O fluxes (*f*N_2_O and *f*CH_4_) by applying the following equation [25]:

$$Flux\;\left(F\right)=\left(\frac{S\times V}{A}\right)\times a_{1}\times a_{2}$$ (1)

where *S* is the slope (ppm s^-1^), *V* is the chamber volume (m^3^), *A* is the chamber area (m^2^), *a*_1_ (86400 s d^-1^) is a conversion factor from seconds to days, and *a*_2_ (0.6788 mg m^-3^ and 1.8625 mg m^-3^ for CH_4_ and N_2_O, respectively) is a conversion factor from ppm to mg m^-3^ at *in-situ* measured temperature (*T* in K) and standard pressure (*p* in Pa):

$$a_{2}=\frac{M\times P}{8.31\;{J∙\mathrm{mol}}^{-1}∙K^{-1}\times T}$$ (2)

where *M* is the molar mass of CH_4_ and N_2_O (g mol^-1^).

Fluxes of N_2_O and CH_4_ from each tank in both plants were used to estimate emission factors (EF) which expressed by kg N and C emitted as N_2_O and CH_4_ per kg DIN and COD in influent wastewater. These factors provide information about the influence of the operational procedures and process design on the mass balance approach over each treatment stage.

### Analysis of isotopocule ratios

For analysis of isotopocule ratios of DN_2_O, samples were prepared by injecting a headspace of 120 mL of ultrapure helium (He) and subsequent equilibrating of liquid and gas phases at constant temperature (20°C). The headspace was transferred into 115 mL glass bottles, which had been flushed with N_2_ gas. The analyses were performed using a Delta XP isotope ratio mass spectrophotometer (IRMS, MAT 251, Thermo–Finnigan, Bremen, Germany), which allows simultaneous detection of m/z 30, 31, 44, 45 and 46. The notation of the isotopocule ratios is the following:

$$\delta^{15}N^{i}={(}^{15}R^{i}{}_{sample}{/}^{15}R^{i}{}_{std}-1)\times 1000(‰)\;(i‑\alpha ,\;\beta \;\mathrm{or}\;\mathrm{bulk})$$ (3)

$$\delta^{18}O={(}^{18}R_{sample}{/}^{18}R_{std}-1)\times 1000(‰)\;(i‑\alpha ,\;\beta \;\mathrm{or}\;\mathrm{bulk})$$ (4)

where ^15^R^α^ and ^15^R^β^ represent the ^15^N/^14^N ratios of α and β N atoms, respectively. ^15^R^bulk^ and ^18^R denote average isotope ratios for ^15^N/^14^N and ^18^O/^16^O, respectively. Subscripts “sample” and “std”, respectively, signify isotope ratios for the sample and the standard, atmospheric N_2_ for N and Vienna Standard Mean Ocean Water (VSMOW) for O.

The ^15^N site preference (hereinafter, SP) was also defined as an illustrative parameter of the intramolecular distribution of ^15^N. Site-specific N isotope analysis in NO was conducted using ion detectors modified for mass analysis of the N_2_O fragment ions (NO^+^), which contained N atoms in the α position of the N_2_O molecules, whereas bulk (average) N and O isotope ratios were determined from molecular ions (N_2_O^+^) [19]:

$${}^{15}N-site\;preference\;\left(SP\right)=\delta^{15}N^{\alpha }-\delta^{15}N^{\beta }$$ (5)

SP values for different production pathways (hydroxylamine (NH_2_OH) oxidation by bacterial nitrification, NO_2_^-^ reduction during bacterial denitrification, nitrifier-denitrification and fungal denitrification) can be divided into two ranges. Specifically, NH_2_OH oxidation by bacterial nitrification (SP is 27.2‰-35.6‰,)[26] and NO_2_^-^ reduction by fungal denitrification (SP is 34.1‰-39.6‰; [27]) show higher SP values whereas denitrification conducted by bacteria (nitrifier and denitrifier) shows lower SP (bacterial denitrification: SP is -6.9‰ to 1.4‰; [26,28]; nitrifier-denitrification: SP is -13.6‰ to 5.0‰; [29,30,31]).

Assuming the absence of N_2_O reduction, the contributions of NO_2_^-^ reduction (*x*) and NH_2_OH oxidation (*1 –x*) to N_2_O production can be estimated from SP:

$$SP_{sample}=xSP_{NO_{2}{}^{-}reduction}+(1-x)SP_{NH_{2}OHoxidation}$$ (6)

Therein, SP_NO2- reduction_ and SP_NH2OH oxidation_, respectively, denote the SP values when N_2_O is produced only by NO_2_^-^ reduction and when N_2_O is produced only by NH_2_OH oxidation.

The δ^15^N of NH_4_^+^ was measured using the diffusion method [32] where about 10 μmol of NH_4_^+^ in the sample was concentrated onto a GF/D glass fiber filter containing H_2_SO_4_ and analyzed using an EA1110 elemental analyzer (Thermo Fisher Scientific K.K.) coupled with the IRMS.

## Results and discussion

### N_2_O emissions

#### Characteristics of dissolved inorganic nitrogen (DIN) compounds and DN_2_O

**Fig 2:**
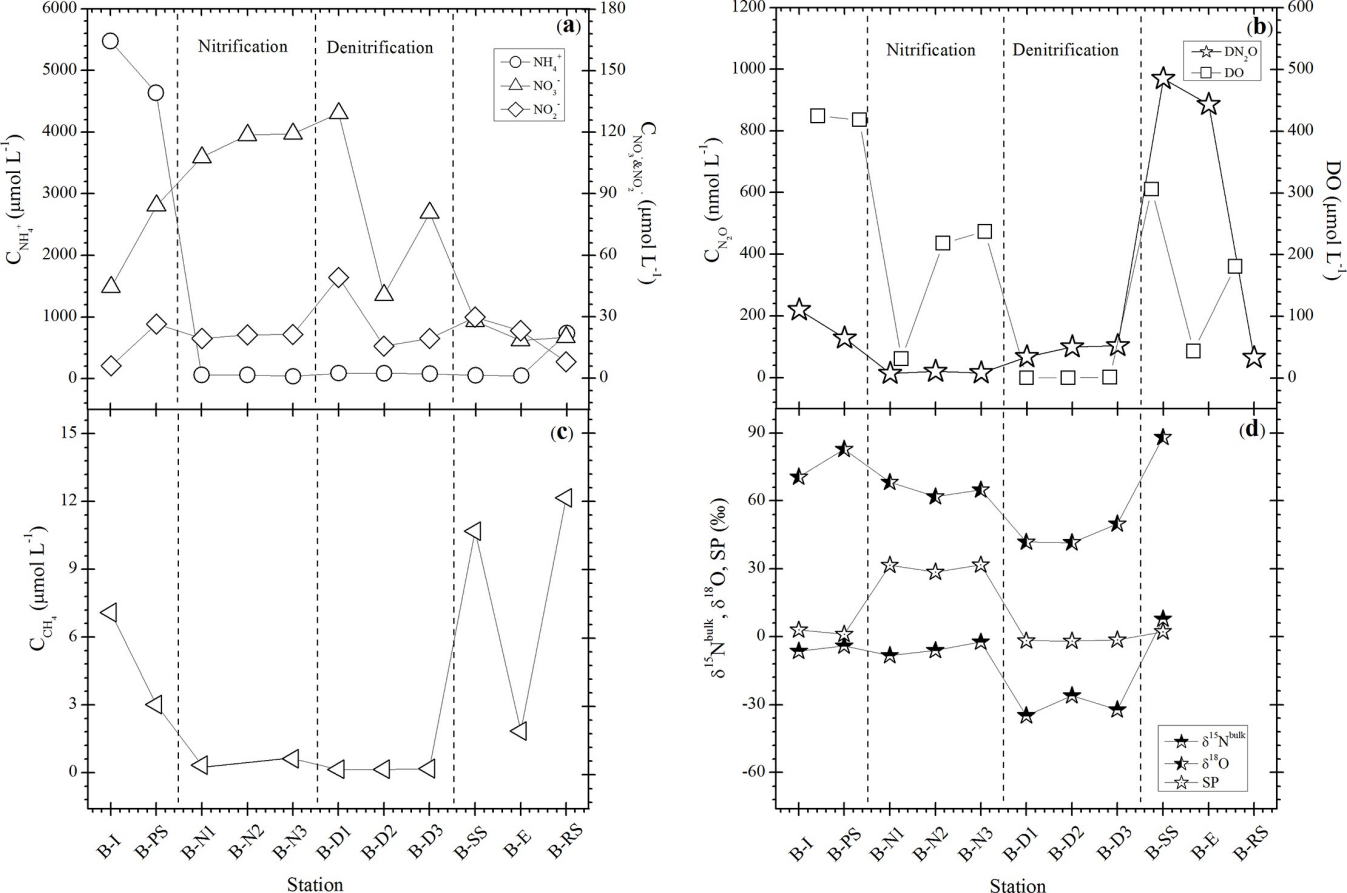
Concentration of dissolved inorganic nitrogen species (a) N_2_O, DO (b), CH_4_ (c) and isotope/isotopocule ratios (d) of N_2_O at each station in the CAS system at Bellheim.

**Fig 3:**
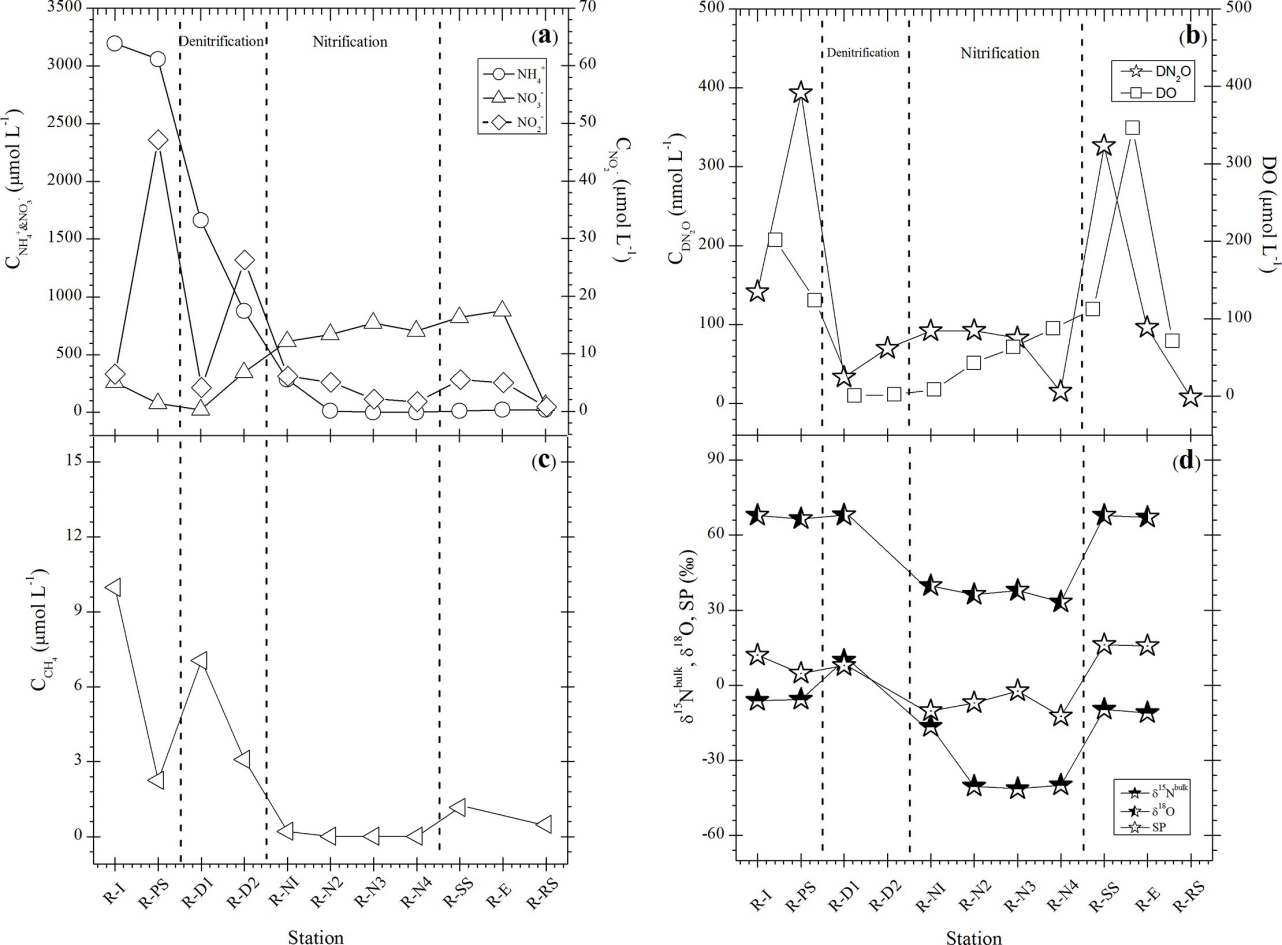
Concentration of dissolved inorganic nitrogen species (a) N_2_O, DO (b), CH_4_ (c) and isotope/isotopocule ratios (d) of N_2_O at each station in the MLE system at Ruelzheim.

The distribution of DIN species (NH_4_^+^, NO_2_^-^ and NO_3_^-^), DN_2_O and DO in the water from different sampling points at both WWTPs are presented in Figs 2A, 2B, 3A and 3B, respectively. The concentration of NH_4_^+^ in the influent at both plants decreased rapidly from 5478.6 to 49.1 μmol L^-1^ in Bellheim and from 3192.8 to 22.7 μmol L^-1^ in Ruelzheim, whereas the concentrations of NO_2_^-^ and NO_3_^-^ increased monotonically throughout the treatment process. This confirms the presence of ammonia-oxidizing bacteria (AOB), and nitrite oxidizing bacteria (NOB) as well as ammonia oxidizing archaea (AOA) in the activated sludge. AOA has stronger environmental adaptability than AOB, which provides the possibility for the development of novel nitrogen removal processes with ammonia oxidation dominated by AOA under low oxygen level ([33], Figs 2A and 3A). The build-up of NO_2_^-^ at B-D1 (about 49.1 μmol L^-1^) in Bellheim and at R-D2 (26.3 μmol L^-1^) in Ruelzheim was accompanied by slightly increased DN_2_O and agrees with observations in a previous study, which found that high NO_2_^-^ concentration during denitrification leads to a lower denitrification rate and accumulation of NO and N_2_O [34]. Approximately 98.2% (Bellheim) and 91.6% (Ruelzheim) of influent NH_4_^+^ were removed as shown by mass balance estimation while the majority of the removed N was probably converted into gaseous forms.

DN_2_O concentrations in the nitrification basin in Bellheim were almost constant and ranged between 13.7 and 20.1 nmol L^-1^ at the points from B-N1 to B-N3, where the DO concentrations were between 0.5 (31.3 μmol L^-1^) and 3.8 mg L^-1^ (237.5 μmol L^-1^). N_2_O production is closely linked to oxygen concentration, which plays a critical role in influencing N_2_O emission. This is proved by the obtained results in the nitrification basins in Ruelzheim (R-N1–R-N4), where N_2_O concentrations were relatively high (15.3–92.6 nmol L^-1^), while DO ranged from 0.1 (8.1 μmol L^-1^) to 1.4 mg L^-1^ (87.5 μmol L^-1^). Our results agree with those of Tumendelger et al., [2014] where lower DO was found to increase N_2_O production in the aerobic treatment tank due to local oxygen limitation. In the denitrification basins in Bellheim, N_2_O concentrations increased gradually up to 103.5 nmol L^-1^ in B-D3 at a DO concentration of 0.03 mg L^-1^ (1.9 μmol L^-1^) while DN_2_O increased to 69.9 nmol L^-1^ at R-D2 in Ruelzheim at a comparable oxygen concentration. However, oxygen can inhibit both synthesis and activity of denitrifying enzymes. N_2_O reductase is more sensitive to oxygen than the other enzymes, leading to N_2_O emission during denitrification when oxygen is present even in low amounts [Kampschreur et al., 2009]. Aside from DO, N_2_O production can depend on the carbon to nitrogen (C/N) ratio as electron donor is considered to be an important parameter for N_2_O accumulation, especially at low C/N ratios [23]. The highest DN_2_O concentrations of 885–970 nmol L^-1^ were observed in the secondary settling tank (B-SS) and in the effluent water (B-E) at Bellheim, which exhibited a three times greater increase than the DN_2_O concentration at same sampling locations in Ruelzheim. DN_2_O concentrations at all stations in both plants were higher than the atmospheric equilibrium concentration of about 12 nmol L^-1^ at 12°C and 13 nmol L^-1^ at 8°C [35]. The water in the biological reaction basins (nitrification and denitrification) was supersaturated with N_2_O by about 800% (Figs 2B and 3B), indicating that the wastewater treatment system is a source of N_2_O to the atmosphere.

In the receiving streams, the effluent discharge had a significant effect on most of the measured physico-chemical parameters and also on the concentration of DIN, DO, DN_2_O (Table 1). In particular, the upstream water had lower DN_2_O concentration (48.6 nmol L^-1^ at B-US), than the water downstream of the effluent discharge location. However, upstream water was saturated with DN_2_O in comparison to atmospheric equilibrium. The extreme changes in DN_2_O concentration of up to a factor of three suggest that effluent-DN_2_O significantly affected downstream concentrations and that additional amounts of N_2_O produced during the treatment process are released to atmosphere from the effluent-receiving streams. The large difference between the DN_2_O concentrations in the effluents of both WWTPs is most likely related to treatment operation conditions and capacity. The NO_3_^-^ concentration was elevated up to 402.1 μmol L^-1^ in R-DS and 72.1 μmol L^-1^ in B-M. Basically, high NO_3_^-^ can be presented due to (1) denitrification occurrence in the stream sediment, (2) nitrification in oxygen-rich stream water (14), DN_2_O was increasing simultaneously. However, the interpretations based on concentration measurements are further examined using the stable isotopic analysis described below.

**Table 1:** Summary of measured parameters in the receiving streams before effluent addition (upstream, US), at the mixing point (M) and downstream (DS) of the WWTP.

Plant	Rulzheim			Bellheim		
Sampling point	upstream R-US	mixing R-M	downstream R-DS	upstream B-US	mixing B-M	downstream B-DS
Dissolved O_2_ (mmol L^-1^)	641.88	641.88	nm	893.75	637.50	693.75
Dissoved CH_4_ (mmol L^-1^)	0.42	0.19	nm	0.19	2.44	2.62
Dissolved N_2_O (nmol L^-1^)	56.80	82.90	nm	48.60	101.50	161.70
d^15^N^bulk^-N_2_O (‰)	-2.80	-6.10	-6.10	0.35	1.95	0.96
d^18^O-N_2_O (‰)	71.95	69.36	69.80	69.01	72.03	73.31
SP-N_2_O (‰)	13.10	13.20	12.90	24.35	28.35	26.10
NH_4_^+^ (mmol L^-1^)	9.14	4.64	5.86	23.64	47.0	43.29
NO_2_^-^ (mmol L^-1^)	3.36	2.86	3.50	1.71	28.79	25.79
NO_3_^-^ (mmol L^-1^)	254.30	351.40	402.10	18.57	72.14	69.29
Water temperature (°C)	7.80	8.50	nm	nm	5.50	5.40
pH	8.0	7.80	nm	8.30	7.90	8.0
EC (mS cm^-1^)	860.0	894.0	nm	390.0	796.0	837.0

nm: not measured

#### N_2_O source partitioning based on isotopocule ratios

The δ^15^N^bulk^ (average of α and β sites of N atoms in N_2_O molecules), δ^18^O, and SP of DN_2_O for the water samples collected from both plants are shown in Figs 2D and 3D. In Bellheim, δ^15^N^bulk^ was almost constant and ranged between -2.31‰ and -8.29‰ from the influent to the nitrification basin (B-N1‐B-N3), followed by a sharp decrease in the denitrification basins and an increase in B-SS (Fig 2D). The values observed at B-N1‐B-N3 were comparable to the value of -13.5‰ measured in the oxic tank of a Japanese WWTP by Toyoda et at. [2011]. The δ18O showed a wide range of variation between +41.3‰ and +82.8‰ throughout the treatment processes, however, the general trend was similar to that of the δ15N. The observed decrease in δ15N^bulk^ and δ^18^O could be interpreted as an isotope effect in microbial N_2_O production during the treatment process. We found high SP values (+28.5‰‐ +31.6‰) at B-N1‐B-N3 whereas it was slightly negative (-1.8‰‐ -1.9‰) in B-D1‐B-D3 (Fig 2D). The SP of DN_2_O depends on (1) the symmetry of the intermediate species and (2) the site specificity in N–O bond breakage of the intermediate. The positive SP values suggest that the intermediate is common to both NH_2_OH oxidation and NO_2_^-^ reduction pathway. Its molecular structure must be symmetric, possibly being a free species such as hyponitrite (^-^ONNO^-^), because substrates and active sites are homogeneously distributed in reaction mixtures and no conformational limitation is expected. Then, intramolecular isotopic fractionation during N–O bond breakage can account for the positive SP: cleavage of ^14^N–O bond is preferred over ^15^N–O bond according to kinetic isotope effect [28].

The negative SP values imply that intermediate species have an asymmetric structure and that a specific N–O bond breaks. For instance, we can consider a reaction mechanism in which coordination of NO molecules to Fe center is followed by invasion of another NO [36]. Therefore, SP would be negative because of (1) ^15^NO bounds to Fe more preferentially than ^14^NO due to intermolecular isotope fractionation and (2) the O atom in the first NO molecule is abstracted as H_2_O.

**Fig 4:**
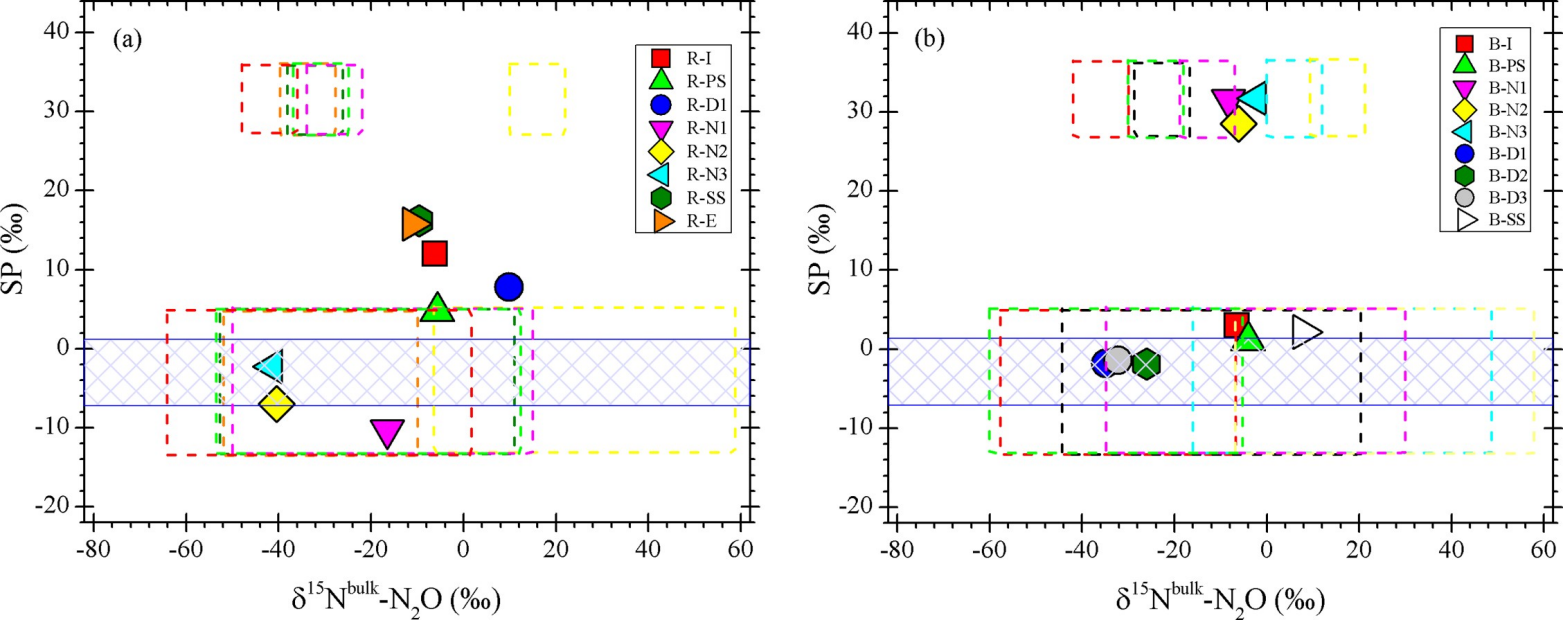
Correlations between SP and δ^15^N^bulk^ of dissolved N_2_O in wastewater from Ruelzheim (a) and Bellheim (b), respectively. Expected ranges for N_2_O produced via NH_2_OH oxidation and NO_2_^-^ reduction calculated according to Toyoda et at. [2005] with the δ^15^N of NH_4_^+^ and the reported isotope effects for each process and corresponding SP are marked by boxes in different colors. We applied the enrichment factors during NH_4_^+^ oxidation to N_2_O (ε^15^N_NH4+→N2O_) of –60 to –48‰ and NO_2_^-^ reduction from NH_4_^+^ to N_2_O (ε(^15^N)_NH4+→NO2-→N2O_) of –76 to –11‰. For N_2_O produced by NO_2_^-^ reduction (denitrification) in samples taken from denitrification basin (blue color), δ^15^N of NO_3_^-^ could not be estimated due to lack of substrate isotope ratio measurement. The SP of N_2_O produced by NH_2_OH oxidation was assigned as +27.2‰‐ +35.6‰, whereas those by NO_2_^-^ reduction during nitrifier-denitrification were -13.6‰‐ +5.0‰.

An important advantage of using the SP is its independence on the ^15^N content of the substrates. In fact, the SP values of N_2_O produced by the microorganisms illustrate that SP differs between NH_2_OH oxidation during bacterial nitrification and NO_2_^-^ reduction during bacterial denitrification (see Material and Methods). However, a laboratory incubation experiment revealed that fungi species and AOA can produce N_2_O with high SP values, similar to bacterial nitrification [fungi: 34.1‰-39.6‰, [27]; AOA: 34.1‰, [37]. Therefore, distinguishing N_2_O produced by bacterial nitrification, fungal denitrification or AOA by isotopic analysis remains challenging. With regard to the N_2_O production during ammonia oxidation by AOA, NH_4_^+^ and NO_2_^-^ both contribute equally via hybrid N_2_O formation [38] and SP values of archaeal N_2_O might have a wider range if the hybrid N_2_O formation occurs. Although SP values by AOA were similar to those of N_2_O produced from NH_2_OH oxidation by AOB, isotopic enrichment factors are distinctive. High uncertainty remains with respect to source partitioning of N_2_O by its stable isotopes and addition of enriched isotope tracers can be a much more powerful and quantitative tool in the case of controlled wastewater systems. Nevertheless, in order to reveal the sources of N_2_O in wastewater, stable isotopic analysis remains a promising tool for partitioning bacterial nitrification and denitrification. N_2_O isotopic fingerprints in the samples were thus assigned to different areas resulting from the contribution of N_2_O production by NH_2_OH oxidation during bacterial nitrification and NO_2_^-^ reduction during bacterial denitrification presented in SP-δ^15^N^bulk^ schematic map (Fig 4). The range of δ^15^N^bulk^ of N_2_O associated with each experiment was estimated from δ^15^N of NH_4_^+^ (in case of nitrification) and the isotopic enrichment factor (ε(^15^N)_NH4_^+^_-→N2O_ = –60 to –48‰, [39]) was estimated from the following equation.

$$\delta^{15}N_{N_{2}O}=\delta^{15}N_{NH_{4}{}^{+}}{}_{\left(NO_{2}{}^{-}\right)}+\varepsilon {{(}^{15}N)}_{substrate\to N_{2}O}$$ (7)

Note that we did not measure the δ^15^N-NO_2_^-^ for the nitrifier-denitrification process. Thus, the δ^15^N^bulk^ was estimated using δ^15^N-NH_4_^+^ and ε(^15^N)_NH4+→NO2-→N2O_. The value is reported as –76 to –11‰ by Toyoda et al., [2011]. The SP values observed at B-N1‐B-N3 were near the range of NH_2_OH oxidation according to the mapping approach, which suggests that NH2OH oxidation during bacterial nitrification was the dominant source of N_2_O in the nitrification basin. During bacterial nitrification, the δ^15^N^bulk^ value showed a gradual decrease with increasing DN_2_O, which could be explained by the production of isotopically light N_2_O. In addition, dominant production of N_2_O in this basin can also be confirmed by the strong decrease in NH_4_^+^ and the accumulation of NO_3_^-^ and NO_2_^-^ (Fig 2A). The contribution of the NH_2_OH oxidation pathway to N_2_O production in this basin was about 86.3‐96.1% (Eq 6). In contrast, the data at B-D1‐B-D3 was falling in the range of NO_2_^-^ reduction, suggesting that the dominant source of the produced N_2_O in the denitrification basin was NO_2_^-^ reduction by bacterial denitrification (Fig 4). Moreover, the lower SP values observed at B-I and B-SS are within the range of the NO_2_^-^ reduction source, indicating that the NO_2_^-^ reduction pathway was dominant (>90%).

In Ruelzheim, N_2_O sampled in the denitrification basin had a greater δ^15^N^bulk^ (9.9‰) than N_2_O produced in the nitrification basin where SP was the lowest and ranged between -12.5‰ and -10.7‰ (Fig 3D). This negative SP values can be caused by the inorganic N_2_O production via NO_2_^-^ reduction (nitrifier denitrification) by Fe^2+^ where SP ranged from -13.3 to +22.6‰ in the measurements of Samarkin et al. [2010] [40].

Relatively few measurements of isotopocule ratios of DN_2_O in fresh water including river and streams have been reported to date [14,24]. In our samples, stream-emitted N_2_O has lower δ^15^N^bulk^ values (+0.35‰—+1.95‰ at Bellheim and -6.1‰—-2.8‰ at Ruelzheim) than tropospheric N_2_O (δ^15^N: +6.72%), indicating that biological N_2_O production is from additional “light” N_2_O in sewage plants. In contrast, the δ^18^O was relatively high about 70‰ in both streams. High δ^18^O values (> 30‰) in rivers or streams are likely produced from denitrification and/or N_2_O consumption which is expected to dominate riverine N_2_O production [15]. In case of upstream (B-U), this may also receive significant N_2_O inputs from ground water that can have high δ^18^O values (see Table 1). For the first time, we report measurements of the SP-N_2_O in small streams nearby WWTPs. The SPs observed at all in-stream sampling sites (+24.3 ‐ +28.3‰) near to Bellheim were comparable to that of the N2O produced via NH_2_OH oxidation by AOB. This production pathway was also suggested by the NO_3_^-^ concentration at the sampling sites (Table 1). At Ruelzheim, in contrast, NO_2_^-^ reduction pathway dominantly contributed to the production of the N_2_O sampled in the streams, with SP values ranging between +12.9 and +13.2‰. Moreover, N_2_O reduction at sampling these sites might be the cause of increased SP (Table 1).

#### Atmospheric fluxes of N_2_O

The amounts of N_2_O emitted to the atmosphere from both WWTPs are presented in Table 2. The total flux from Ruelzheim, which has separate basins for nitrification and denitrification, was 12.1 g-N d^-1^ that converts into an emission factor of 0.008% of N removed from influent DIN and released as N_2_O. Approximately 84% of the total N_2_O emission (10.2 g-N d^-1^) was from the nitrification basin where relatively low DO concentration existed. This high emission could be explained by the oxygen concentration, which is required for the oxidation processes. Because AOB have a higher oxygen affinity than NOB, low oxygen concentration resulted in NO_2_^-^ accumulation. For instance, the combination of low oxygen with elevated NO_2_^-^ accumulation can induce N_2_O production by the nitrifier-denitrification pathway [41]. The end product of this pathway is N_2_O since the genes encoding N_2_O reduction to N_2_ are not yet found. The total flux of N_2_O from Bellheim (3.67 g-N d^-1^, emission factor of 0.001%) was three times lower than that of Ruelzheim. About 76.8% of total N_2_O flux was emitted from the nitrification basin and this high emission rate could be caused by stripping and does not reflect the local production rate of N_2_O. Assuming that if emission and production were not linked, most of the produced N_2_O would remain in dissolved form in the water, and it would be stripped as soon as the aerators are switched on. Our results agreed with Mampaey et al. [2015] who found a strong increase in emission followed by low value as the liquid gets depleted of N_2_O [42]. IPCC [2006] reported that the standard N_2_O emission factor is 3.2 g-N person^-1^ year^-1^ [43], corresponding to approximately 0.035% of the nitrogen load of a WWTP based on first measurement by Czepiel et al. [1995]. Our estimated data at both plants were lower than the IPCC base, and the emission factor observed at Bellheim was similar to Suemer et al. [1995] who obtained a value of 0.001% for activated sludge plant (Table 3) [44]. We found that the emission factors among different WWTPs are highly variable, which could be caused by (1) different treatment capacity and/or (2) different operational parameters including DO concentration, switching time between nitrification and denitrification and so on. Therefore, more research is required to reveal the dependence of the emission factor on operational parameters.

**Table 2:** Estimated fluxes emitted from WWTPs and nearby receiving streams and corresponding emission factors.

Plant	Sample ID	Sampling point	N_2_O flux (mg-Nm^-2^d^-1^)	N_2_O flux (mg-Nd^-1^)	Total N_2_O flux (g-Nd^-1^)	Emission factor, %	CH_4_ flux (mg-Cm^-2^d^-1^)	CH_4_ flux (mg-Cd^-1^)	Total CH_4_ flux (g-Cd^-1^)	Emission factor, %
Rulzheim WWTP (Modified Ludzack Ettinger process with anaerobic digestion system)	R-D1	Denitrification (D1)	3.731	231.250	12.077	0.008	228.653	14171.911	118.077	0.004
	R-D2	Denitrification (D2)	1.657	102.679			37.481	2323.086		
	R-D3	Denitrification (D3)	nm	102.679^a^			nm	2323.086^a^		
	R-D4	Denitrification (D4)	nm	102.679^a^			nm	2323.086^a^		
	R-N1	Nitrification (N1)	7.278	1275.357			34.446	6036.030		
	R-N2	Nitrification (N2)	17.222	3017.727			34.446	6036.030		
	R-N3	Nitrification (N3)	16.853	2953.187			36.846	6456.454		
	R-N4	Nitrification (N4)	16.853	2953.187			nm	6456.454		
	R-SS	Secondary settling	1.724	1338.631			92.667	71950.603		
	R-US	upstream	-0.832				1.536			
	R-DS-M	mixing	0.024				2.631			
	R-DS-50	downstream	0.061				0.261			
Bellheim WWTP (Conventional activeated sludge system)	B-N1	Nitrification (N1)	nm	172.349^b^	3.671	0.001	nm	450.470^b^	373.288	0.01
	B-N2	Nitrification (N2)	0.112	172.349			0.293	450.470		
	B-N3	Nitrification (N3)	1.616	2479.858			0.768	1178.880		
	B-D1	Denitrification (D1)	nm	4.917^b^			nm	1315.048^b^		
	B-D2	Denitrification (D2)	0.003	4.917			0.857	1315.048		
	B-D3	Denitrification (D3)	0.490	751.899			4.439	6813.145		
	B-SS	Secondary settling	0.151	85.502			637.831	361764.987		
	B-US	upstream	2.731				0.267			
	B-DS-M	mixing	1.665				1.569			
	B-DS-50	downstream	1.567				1.814			

nm: not measured

^a^Estimated with the assumption that N_2_O concentration was equal to previous section

^b^Assumed that N_2_O concentration is identical as next section

**Table 3:** Nitrous oxide (N_2_O) emission factors reported for several full-scale wastewater treatment plants.

Process type/Location	N_2_O emission (% of N-load)	Remarks	Reference
Activated sludge plant, USA	0.035	N_2_O emission from aerated zones	Czepiel et al., 1995
Activated sludge plant, Germany	0.001	N_2_O emission increased with NO_2_^-^ and NO_3_^-^ concentrations	Suemer et al., 1995
Activated sludge plant, Japan	0.01–0.08	N_2_O emission decreased with shorter aeration period	Kimochi et al., 1998
Nitritation-anammox sludge water treatment, Netherlands	2.3	N_2_O emission increased with low oxygen concentration (aerated stage) and high nitrite concentration (anoxic stage)	Kampschreur et al., 2008b
Activated sludge plant, USA	0.01–1.8	N_2_O emission increased with high nitrate and dissolved oxygen concentrations (anoxic zones)	Ahn et al., 2010
Activated sludge plant, UK	0.036	N_2_O emission increased with low oxygen concentration	Aboobakar et al., 2013
Activated sludge plant, Denmark	0.15–4.27	N_2_O emission observed under the sub-optimal operation of biological treatment processes	Yoshida et al., 2014
Activated sludge plant, Finland	0.02–2.6	N_2_O emission related to diurnal and long-term variation	Mikola et al., 2014
Conventional activated sludge (CAS) plant, Japan	0.03–0.14	Under different dissolved oxygen concentration (1.5–2.5 mg L^-1^)	Tumendelger et al., 2014
CAS plant, Netherlands	2.8	N_2_O emission occurred in sub-optimal oxygen concentrations	Daelman et al., 2015
CAS plant, Germany	0.001	Most of N_2_O emitted from nitrification basin where dissolved concentration was low	This study
Modified Ludzack-Ettinger (MLE) system with energy recovery, Germany	0.008	N_2_O emission caused by streeping	This study

It can be assumed that all N_2_O dissolved in the effluent water will be released to the atmosphere from the stream. In the stream nearby Ruelzheim, an unexpected negative flux (-0.83 mg N m^-2^ d^-1^), i.e. N_2_O uptake by the stream, was measured at the upstream sampling site (R-US), which can probably be (I) an artifact resulting from relatively short chamber deployment duration or (II), there was some residual organic carbon that may contributed to denitrification or N_2_O consumption that has not been discussed in this study [45]. However, a positive flux of 0.02 mg N m^-2^ d^-1^ was observed at mixing point (R-DS-M). The most likely explanation for the flux observed at this point relates to higher contribution of effluent-N_2_O from the plant (Table 3).

### CH_4_ emission

#### Characteristic of DCH_4_

All water samples were supersaturated with CH_4_ in respect to atmospheric equilibrium and dissolved gas concentrations varied widely among sampling sites (Figs 2 and 3). DCH_4_ ranged between 0.14 and 12 μmol L^-1^ (average = 3.2±4 μmol L^-1^) and 0.01 and 9.9 μmol L^-1^ (average = 2.2±3 μmol L^-1^) at Bellheim and Ruelzheim WWTP, respectively. We did not observe a significant correlation between DO and DCH_4_ at the sampling sites at Bellheim WWTP, suggesting that large portions DCH_4_ were not produced at the sampling sites, but delivered from the preceding treatment steps. In contrast, we observed a correlation between DO and CH_4_ for treatment processes at Ruelzheim indicating that CH_4_ existence at these sites may be linked to local production. DCH_4_, and DCO_2_ did not differ significantly between the nitrification and denitrification basins at both WWTPs (ANOVA, *p* < 0.05). High concentrations of dissolved CH_4_ were observed in the inflows of both WWTPs (Fig 2 and Fig 3), indicating that considerable amount of CH_4_ is delivered to the plants prior the treatment. High concentrations declined rapidly during the further treatment process, suggesting that a high percentage of the dissolved CH_4_ is emitted during the mechanical motion of the wastewater at primary settling systems. Approximately 60% of the dissolved CH_4_ in the inflowing wastewater was released at the primary settling at both WWTPs. However, there were no flux measurements at these points. Consistent and relatively low DCH_4_ concentrations were observed during the nitrification processes (N1, N2 and N3) at both plants. High DCH_4_ values were observed in the denitrification basins of Bellheim WWTP, which could be caused by the low oxygen concentration. DCH_4_ increased in the secondary clarifier at both plants, where it was potentially produced under anaerobic conditions in the settled sludge.

The discharge of effluent water caused a 13-fold increase of DCH_4_ in the receiving streams at Bellheim, while the concentration decreased in Ruelzheim (Tabel 1). WWTPs have been shown to export additional amount of DCH_4_ and this study confirmed the potential importance of WWTP effluents on the concentration of CH_4_ in inland water [13]. More studies are required for investigation the factors controlling the magnitude and variability of DCH_4_ exported with effluent water from WWTPs.

#### Atmospheric fluxes of CH_4_

Most sampling sites (16 out of 18 flux measurements), were net sources of atmospheric CH_4_ (Table 2). In accordance with the high concentrations of DCH_4_, high fluxes were observed at the denitrification basins and secondary settlers at both plants. At the secondary settlers, fluxes were additionally enhanced by the high gas exchange rate caused by the mechanical motion of skimmer arm and associated turbulence at the water surface. Based on our measurements, the nitrification basins are hot spots of CH_4_ emission. In contrast to the denitrification process, the nitrification process is expected to have a high gas exchange velocity caused by air injection. The flux measurements are subject to uncertainty caused by spatial and temporal variability of flux rates, which were not resolved in the single point measurements. Additional uncertainty of the flux measurements at the nitrification/denitrification basins are related to the chamber effect on the surface turbulence [46], as the chambers were anchored with the frame of the basins, while the chambers were freely floating at the other different sites.

Our measurements in Ruelzheim resulted in a total flux of 118.1 g-C d^-1^ as CH_4_ or an emission factor of 0.004% of influent oxidized COD. Approximately 60.9% of the total CH_4_ emission (71.9 g-C d^-1^) occurred at the secondary settler while 21.1% (25.0 g-C d^-1^) were emitted from the nitrification basin and 7% from the denitrification basin. At Bellheim, the total flux of CH_4_ was 373.3 g-C d^-1^, corresponding to an emission factor of 0.01%, which is about three times greater than the emission factor at Ruelzheim. Most CH_4_ was emitted from the denitrification and secondary settling tanks.

Only few estimates of CH_4_ emission factors exist. Czepiel et al. [1993], Wang et al. [2011] and Daelman et al [2012] estimated emission factors ranging between 0.08−1.13% of the COD load [1,8,47]. The Dutch Ministry of Housing, Spatial Planning and the Environment in the Netherlands presents estimation results in a CH_4_ emission factor of 0.85% for WWTPs with anaerobic sludge treatment while it was 0.70% for plants without anaerobic sludge treatment [1]. Our estimated data at both plants were lower than the emission factors reported in the above-mentioned studies. We expect that considerable amounts of DCH_4_ are oxidized prior to discharge. However, high spatial and temporal measurement resolution is an urgent need for investigating production, emission and oxidation process of CH_4_ during wastewater treatment process.

## Conclusions

A flux of 12.1g-N d^-1^ was emitted as gaseous N_2_O from the MLE system, while it was 3.67g-N d^-1^ from CAS system. In contrast, the CAS system had a higher CH_4_ emission factor than the MLE system. SP-N_2_O suggested that 86.3‐96.1% of N_2_O was produced by NH_2_OH oxidation in the CAS system, whereas nitrifier-denitrification was the major N_2_O production pathway in the same basins in the MLE system. Effluent from the WWTPs increased dissolved gas concentrations and fluxes in the effluent-receiving streams. Microbial denitrification and NH_2_OH oxidation mainly produced N_2_O in the receiving streams nearby WWTPs as revealed by SP.
